# Supplementary material for: Optimization of Protoplast Preparation Conditions in Lyophyllum decastes and Transcriptomic Analysis Throughout the Process
Source: J Fungi (Basel). 2024 Dec 21;10(12):886. doi: 10.3390/jof10120886 (PMC11678723; doi:10.3390/jof10120886)
Supplement: Supplementary file 1 [file jof-10-00886-s001.zip › Scheme S1.pdf]

Scheme S1: Original Protocol for Transcriptome sequencing, assembly, DEG functional annotation and qRT--PCR validation.

RNA Extraction and Sequencing

Total RNA was isolated using the Trizol Reagent (Invitrogen Life Technologies), after which the concentration, quality, and integrity were determined using a NanoDrop spectrophotometer (Thermo Scientific). Sequencing libraries were generated using the TruSeq RNA Sample Preparation Kit (Illumina, San Diego, CA, USA). mRNA was purified from total RNA using poly-T oligo-attached magnetic beads. Then the enriched mRNA was fragmented into short fragments using a fragmentation buffer and reverse transcribed into cDNA with random primers. The library fragments were purified using the AMPure XP system (Beckman Coulter, Beverly, CA, USA). DNA fragments with ligated adaptor molecules on both ends were selectively enriched using Illumina PCR Primer Cocktail. Products were purified (AMPure XP system) and quan-tified using the Agilent high-sensitivity DNA assay on a Bioanalyzer 2100 system (Ag-ilent). The sequencing library was then sequenced on a NovaSeq 6000 platform (Illu-mina) by Shanghai Personal Biotechnology Co., Ltd.

De novo Transcriptome Assembly and Gene Annotation

Samples are sequenced on the platform to get image files, which are transformed by the software of the sequencing platform, and the original data in FASTQ format (Raw Data) is generated. Sequencing data contains a number of connectors and low-quality reads, so we use fastp (v0.22.0) software to filter the sequencing data to get high-quality sequences (Clean Reads) for further analysis. For the transcriptome se-quencing project without a reference genome, we use Trinity (v2.15.1) software to assemble Clean Reads for transcripts for later analysis. After the completion of assem-bly, transcript sequence files in FASTA format can be obtained. The longest transcript of each gene was extracted as the representative sequence of the gene, called Unigene. We have annotated gene functions for Unigenes. The databases used in gene function annotation include NR (NCBI non-redundant protein sequences), GO (Gene Ontology), KEGG (Kyoto Encyclopedia of Genes and Genomes), eggNOG (evolutionary genealogy of genes: Non-supervised Orthologous Groups), Swiss-Prot, and Pfam.

Differential Gene Expression Analysis and Functional Enrichment

Using RSEM (v2.15) statistics, we compared the Read Count values for each gene to represent its original expression level, and FPKM was used to normalize gene ex-pression levels. Three pairwise comparisons were conducted using RNA-seq data, spe-cifically between LDY1 vs. LDY2, LDY2 vs. LDY3, and LDY1 vs. LDY3. Next, we used DESeq (v1.38.3) to analyze the differentially expressed genes (DEGs), with screening criteria of $| \log2FoldChange \; | > 1$ and a significant P-value < 0.05. All DEGs were mapped to terms in the Gene Ontology database, and the number of enriched DEGs was calcu-lated for each term. GO enrichment analysis was performed using topGO (v2.50.0), with P-values calculated through the hypergeometric distribution method, where sig-nificant enrichment was defined by a P-value < 0.05. This enabled the identification of GO terms with significantly enriched DEGs, thereby determining the primary biologi-cal functions of these genes. KEGG pathway enrichment analysis of DEGs was per-formed using ClusterProfiler (v4.6.0), focusing on significantly enriched pathways (P-value < 0.05).

Quantitative real-time polymerase chain reaction (qRT-PCR) verification

To validate the RNA-seq results, 11 DEGs were selected to evaluate the consisten-cy of their expression patterns. Total RNA was extracted using Trizol Reagent (Invitro-gen Life Technologies) and reverse-transcribed into cDNA using the PrimeScriptTM 1st Strand cDNA Synthesis Kit. Glyceraldehyde 3-phosphate dehydrogenase (GAPDH) served as the housekeeping gene [1]. The reaction system consisted of 10 μl of 2× SYBR real-time PCR premixture, 0.4 μl of each forward and reverse primer, and 1 μl of cDNA, with RNase-free dH2O added to reach a total volume of 20 μl. The qRT-PCR program was configured as follows: 95°C for 5 minutes, followed by 40 cycles at 95°C for 15 seconds and 60°C for 30 seconds. Each

sample was analyzed in triplicate. Rela-tive gene expression levels were determined using the $2^{-\Delta\Delta Ct}$ method [2].

References

1. Hu, Y.; Li, J.; Lin, H.; Liu, P.; Zhang, F.; Lin, X.; Liang, J.; Tao, Y.; Jiang, Y.; Chen, B. Ultrasonic treatment decreases Lyophyllum decastes fruiting body browning and affects energy metabolism. *Ultrason. Sonochem* **2022**, *89*, 106111. https://doi.org/10.1016/j.ultsonch.2022.106111.
2. Livak, K.J.; Schmittgen, T.D. Analysis of relative gene expression data using real-time quantitative PCR and the 2(-Delta Delta C(T)) Method. *Methods* **2001**, *25*, 402–408. https://doi.org/10.1006/meth.2001.1262.
