# Supplementary figures and images for: Optimization of Protoplast Preparation Conditions in Lyophyllum decastes and Transcriptomic Analysis Throughout the Process
Source: J Fungi (Basel). 2024 Dec 21;10(12):886. doi: 10.3390/jof10120886 (PMC11678723; doi:10.3390/jof10120886)

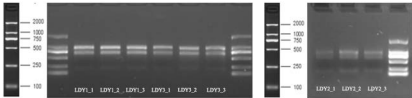

Figure S1 :cDNA agarose gel electrophoresis results.

Supplement: Supplementary file 1 [file jof-10-00886-s001.zip › FIgure S1.pdf]
